# Be Smart to Identify the Stroke-Like Migraine Attacks After Radiation Therapy (SMART) Syndrome

**DOI:** 10.7759/cureus.21930

**Published:** 2022-02-05

**Authors:** Meari Taguchi, Kyle Bonner, Anza B Memon

**Affiliations:** 1 Neurology, Henry Ford Health System, Detroit, USA; 2 Neurology, Wayne State University School of Medicine, Detroit, USA

**Keywords:** migraine, stroke-like episodes, radiation therapy, cancer, brain, smart

## Abstract

Stroke-like migraine attacks after radiation therapy (SMART) are uncommon, often occurring years or decades after brain radiation therapy. This syndrome is a diagnosis of exclusion, and only about 40 cases describing SMART have been published, each one describing a constellation of symptoms and findings. Because symptoms can arise years after initial radiation therapy, the ability of physicians to recognize SMART and rule out other possible causes of symptoms is critical for the long-term care of oncology patients who have undergone cranial radiation. Here we present the case of a 55-year-old man who experienced SMART nine years after radiation therapy and who was successfully treated with steroids.

## Introduction

Stroke-like migraine attacks after radiation therapy (SMART) syndrome was initially described by Shuper et al. in 1995. But it wasn’t until after more cases were published, almost a decade later, that modified diagnostic criteria were proposed by Black et al. [1]. Black’s modified diagnostic criteria emphasize several main characteristics, including that the history of external beam cranial irradiation be “remote". While this term was not specifically defined, the time intervals between radiation treatment and SMART in Black’s case series ranged from 1.2 to 21 years. Other criteria include no evidence of recurrent or residual neoplasm and a lack of other possible attributable disorders [1,2].

SMART may manifest with a range of reversible signs and symptoms that are related to unilateral cortical regions, such as confusion, seizures, aphasia, visual-spatial deficits, hemisensory deficits, headache, or antecedent migraine with or without aura [1,3]. Additional criteria used to identify SMART include the presence of unilateral cortical gadolinium enhancement of the cerebral gyri that is transient and diffuse, not within the white matter, and located within a previous radiation field [1,3].

With advancements in oncology therapies and the extension of oncology patient survival rates, more opportunities for encountering patients with remote effects of cranial radiation therapy, such as SMART syndrome, are possible. SMART syndrome has been reported in approximately 100 case reports, yet no consensus regarding the pathophysiology behind the syndrome or proper treatment exists [3]. In this case report, we describe a patient who presented with visual changes and right-sided weakness nine years after initial radiation therapy for medulloblastoma. This case highlights a diagnostic progression to SMART syndrome that can serve as a template for physicians who may encounter this rare pathology.

## Case presentation

A 55-year-old man presented to the emergency department with mild bilateral occipital headache, right hemiparesthesia, and visual obscurations. On presentation, he reported seeing multicolored shapes including circles, flashes, visual field cuts, and scintillating scotoma. Neurological examination revealed right upper homonymous quadrantanopia, bilateral horizontal nystagmus and dysmetria, hemiparesis, and hyperreflexia of his right extremities. The patient had medulloblastoma in 2011, status post gross total resection, for which he had received three months of radiation therapy.

**Figure 1:**
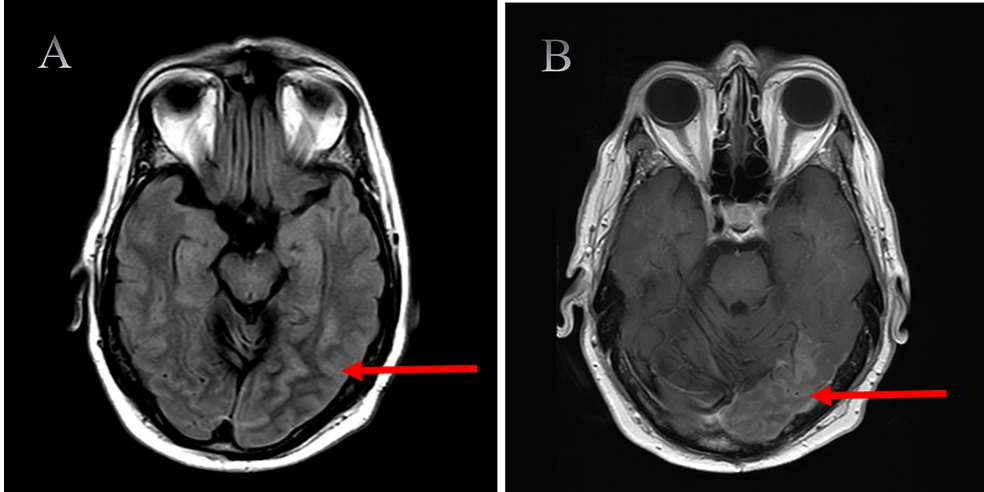
FLAIR axial T2 shows gyral edema in the left parietal and occipital regions (A) with associated gadolinium enhancement in the left occipital region (B) FLAIR: fluid-attenuated inversion recovery

**Figure 2:**
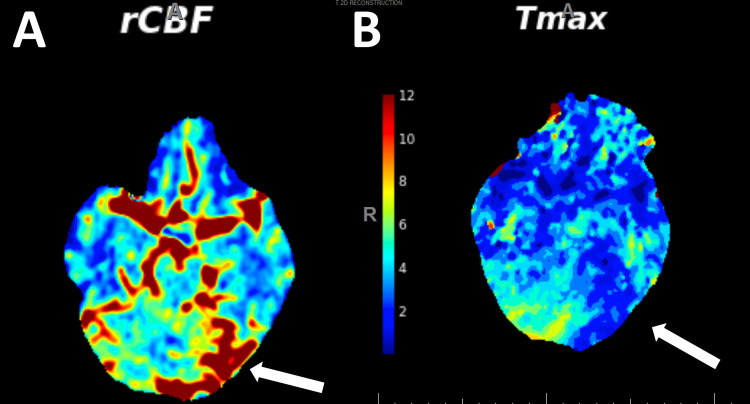
Computed tomography perfusion showing increased cerebral blood flow in the left occipital lobe (A) with correlating reduction in time-to-maximum (Tmax) (B) rCBF: regional cerebral blood flow

**Figure 3:**
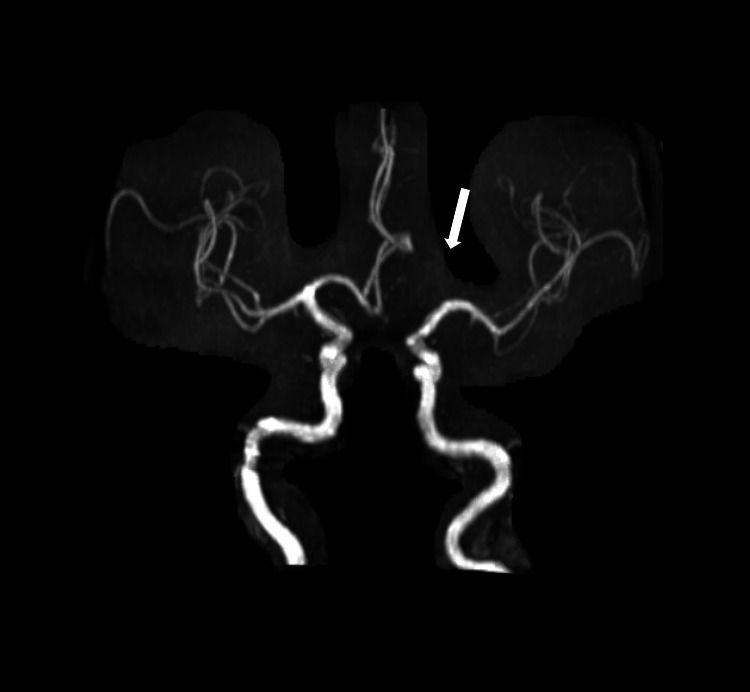
Magnetic resonance angiogram of the head showing an absent or hypoplastic left A1 segment, but otherwise with no large vessel obstruction and no flow-limiting stenosis.

Magnetic resonance imaging (MRI) of the brain revealed gyral edema in the left parietal and occipital lobes on fluid-attenuated inversion recovery (FLAIR) axial sequences, with associated gadolinium enhancement in the left occipital lobe (Figure 1). Computed tomography (CT) perfusion of the head showed increased cerebral blood flow and decreased time-to-maximum (Figure 2). Autoimmune and paraneoplastic panels were negative. Laboratory tests for limbic encephalitis, anti-N-methyl-D-aspartate (NMDA) receptor antibody, and anti-neuromyelitis optica-IgG antibody were also negative. Cerebrospinal fluid had mild protein elevation at 66 mg/dL, but was otherwise negative, including for anti-glutamic acid decarboxylase and anti-myelin oligodendrocyte glycoprotein antibodies (Table 1). Vessel imaging, including CT angiogram and magnetic resonance angiogram, were negative for vasculitis (Figure 3). Routine and video electroencephalograms (EEG) were negative for interictal and ictal discharges. To rule out malignancy, CT of the chest, abdomen, and pelvis was obtained, which indicated a 1.4 cm left exophytic renal cyst. MRI of the abdomen with renal protocol did not indicate concerns for malignancy.

**Table 1:** Supplementary Table Ab: antibody; AchR: acetylcholine receptor; AGNA-1: type 1 anti-glial nuclear antibody; ANA: antinuclear antibody; ANCA: antineutrophil cytoplasmic antibodies; ANNA-1: type 1 antineuronal nuclear antibody; AQP4: aquaporin-4; C-ANCA: antineutrophil cytoplasmic autoantibody, cytoplasmic; CSF: cerebrospinal fluid; CRMP-5: collapsin response-mediator protein-5; ds: double-stranded; DVRTT: dilute Russell viper venom time; EBV: Epstein-Barr virus; GAD: glutamic acid decarboxylase; HSV: herpes simplex virus; IgA: immunoglobulin A; IgG: immunoglobulin G; IgM: immunoglobulin M; MOG: myelin oligodendrocyte glycoprotein; NMO: neuromyelitis optica; P-ANCA: perinuclear anti-neutrophil cytoplasmic antibodies; PCA: Purkinje cytoplasmic antibody; PCR: polymerase chain reaction; S: serum; VRDL: venereal disease research laboratory;

		Labs	Reference Range and Units
CSF	N-methyl-D-Aspartate Receptor Ab IgG Serum with Reflex to Titer	< 1:1	<1:1
	West Nile IgG Abs	< 1.3	<1.30 Antibody not detected 1.30 - 1.49 Equivocal >1.49 Antibody detected
	West Nile IgM Abs	< 0.9	<0.90 Antibody not detected 0.90 - 1.10 Equivocal >1.10 Antibody detected
	Varicella zoster, PCR	Not detected	Not detected
	Cytomegalovirus PCR, Qualitative	Not detected	Not detected
	EBV DNA, PCR CSF	Not detected	Not detected
	HSV 1 DNA	Not detected	
	HSV 2 DNA	Not detected	
	VRDL	Nonreactive	
	Protein	66	15-55 mg/dL
	Lactic Acid	2.4	1.2-2.4 mmol/L
	Glucose	64	40-80 mg/dL
	RBC	<3	0/cu mm
	WBC	3	0-5/cu mm
	Neutrophils	15	0-6%
	Lymphocytes	69	40-80%
	Monocytes	16	15-45%
	Mononucleates	0	15-45%
	Macrophages	0	0%
	Eosinophils	0	0%
	Basophils	0	0%
	AChR Ganglionic Neuronal Ab, S	0.00	= 0.02 nmol/L
	Amphiphysin Ab, S	Negative	<1:240 titer
	AGNA-1, S	Negative	<1:240 titer
	ANNA-1, S	Negative	<1:240 titer
	ANNA-2, S	Negative	<1:240 titer
	ANNA-3, S	Negative	<1:240 titer
	CRMP-5-IgG, S	Negative	<1:240 titer
	Neuronal (V-G) K+ Channel Ab, S	Negative	< 0.02 nmol/L
	N-Type Calcium Channel Ab P/Q-Type Calcium Channel Ab	0.00	< 0.03nmol/L <0.02nmol/L
	PCA-1, S	Negative	<1:240
	PCA-2, S	Negative	<1:240
	PCA-Tr, S	Negative	<1:240
	Striational (Striated Muscle) Ab, S	Negative	<1:240
Serum	ANA Screen and Titer	Negative	Negative
	GAD Ab	<5	< 5 IU/mL
	MOG-IgG1	Negative	Negative
	NMO/AQP4 IgG	Negative	Negative
	Scl-70 Antibodiy	2	<20 units
	B2 Glycoprotein IgG Ab	<9	< 20 SGU
	B2 Glycoprotein IgM Ab	<9	< 20 SMU
	B2 Glycoprotein IgA Ab	<9	< 20 SMU
	Angiotensin Coverting Enzyme	13	8-52 U/L
	SS A/Ro Ab	< 0.2	< 1.0 Elisa Units
	SS B/La Ab	< 0.2	<1.0 Elisa Units
	C-ANCA	< 1:20	<1:20 TIter
	P-ANCA	<1:20	<1:20 Titer
	DRVTT	42	27-45 sec
	Lupus Anticoagulant	38.7 sec	30.3-43..2 sec
	DNA Ab (ds) Crithidia, IFA	Negative	Negative
	Cardiolipin Ab IgA	< 9.0	< 12APL
	Cardiolipin AB IgG	< 9.0	< 15 GPL
	Cardiolipin Ab IgM	< 9.0	< 12.5 MPL
	C4 Complement	48	10-51 mg/dL
	C3 Complement	185	90-230 mg/dL
	AChR Ganglionic Neuronal Ab, S	0.00	= 0.02 nmol/L
	Amphiphysin Ab, S	Negative	<1:240 titer
	AGNA-1, S	Negative	<1:240 titer
	ANNA-1, S	Negative	<1:240 titer
	ANNA-2, S	Negative	<1:240 titer
	ANNA-3, S	Negative	<1:240 titer
	CRMP-5-IgG, S	Negative	<1:240 titer
	Neuronal (V-G) K+ Channel Ab, S	Negative	< 0.02 nmol/L
	N-Type Calcium Channel Ab	0.00	< 0.03nmol/L
	P/Q-Type Calcium Channel Ab	0.00	<0.02nmol/L
	PCA-1, S	Negative	<1:240
	PCA-2, S	Negative	<1:240
	PCA-Tr, S	Negative	<1:240
	Striational (Striated Muscle) Ab, S	Negative	<1:120

**Figure 4:**
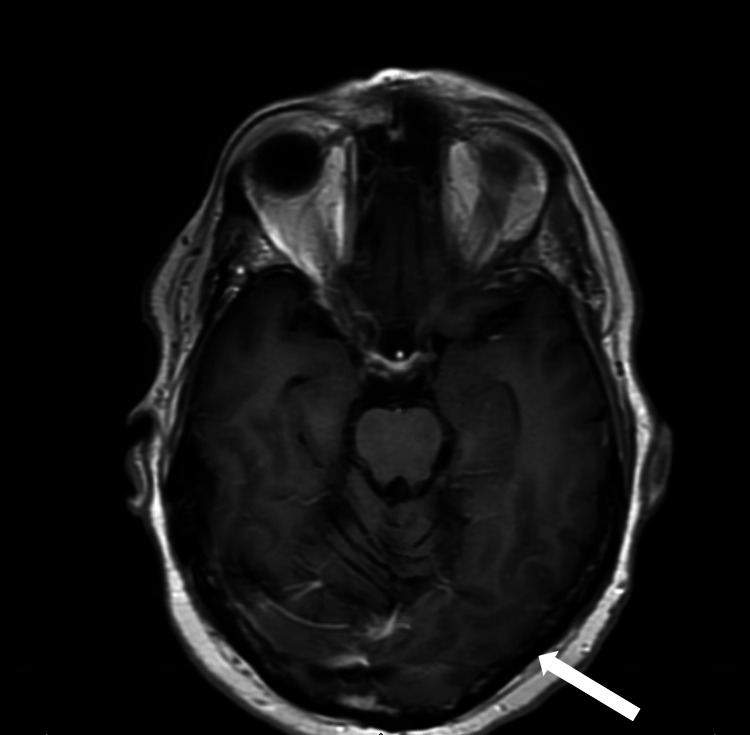
Repeat brain MRI after six months showing interval improvement of the contrast enhancement in the left occipital lobe (white arrow)

The patient was treated with levetiracetam for approximately one month, but the antiepileptic was discontinued after a negative prolonged EEG and when the patient did not demonstrate the improvement of symptoms. While admitted to the hospital, he was treated with a three-day course of 1000 mg intravenous (IV) methylprednisolone daily and oral prednisone 60 mg daily with 20 mg taper every three days. The patient showed complete resolution of his symptoms after two months of therapy. Repeat brain MRI six months after presentation showed resolution of enhancement in the left occipital region (Figure 4).

## Discussion

Here we described a 55-year-old man who had received radiation therapy for medulloblastoma nine years before presenting with symptoms and findings indicating SMART syndrome. Of SMART reported in the literature, the exact pathophysiology and treatment recommendations have not been clearly established. Hypotheses regarding pathophysiology for SMART include radiation-induced endothelial damage leading to impaired cerebrovascular autoregulation [3]. In the literature, the onset of symptoms ranges from one to 35 years after initial radiation [1]. 

Imaging findings in patients with SMART, as in our patient, show a preferential manifestation in the parieto-occipital cortex, suggesting that this region may be more susceptible to damage from radiation therapy [4]. Common imaging findings in cases of SMART include unilateral focal gyral thickening and enhancement, but imaging may also show bilateral effects, including diffusion restriction, cavernoma, and microbleeds [3,4]. MRI findings are generally temporary and typically persist for weeks to months [5]. In patients with epilepsy, MRI findings can be similar to those for SMART syndrome. However, it has been well established that the neurological abnormalities seen in SMART are not due to prolonged metabolic demand as occurs in epilepsy. In patients with SMART who have had seizure-like activity, MRI findings were usually present before the seizure event and were without ictal correlate on EEG [1,2,4]. 

SMART syndrome is a diagnosis of exclusion, and possible causes of symptoms such as seizure disorders, tumor growth/recurrence, infection, posterior reversible encephalopathy syndrome, and stroke must be ruled out. Paraneoplastic and autoimmune syndromes should be considered in patients presenting with symptoms of SMART syndrome since patients suspected of having SMART syndrome have had previous malignancies, which are prone to recurrence [3].

Although SMART is generally a reversible phenomenon, older adults who have temporal lobe involvement or restricted diffusion are more likely to have an incomplete recovery by 10 weeks [3]. Notably, while many patients with SMART have been treated successfully with steroid therapy, as in the case of our patient, steroids are not considered the standard of care, and patients with SMART have recovered without this treatment approach [5]. Ultimately, patients with a history of brain irradiation therapy may be at risk of developing SMART, even many years after initial treatment. Thus, for patients with neurological manifestations that cannot be explained by most common causes, it is critical that physicians ascertain whether the patient has a history of brain irradiation therapy so that the diagnostic hallmarks of SMART can be investigated.

## Conclusions

Here we described a rare case of SMART syndrome in a patient who presented with headaches, visual changes, and right-sided weakness nine years after radiation therapy. After ruling out etiologies such as seizure disorder, infection, tumor recurrence, and neoplastic or paraneoplastic syndromes, our patient was successfully treated with IV steroids. Similar to previously reported courses of SMART syndrome, our patient’s symptoms and imaging abnormalities resolved within a few months. Because of advances in cancer treatment and subsequent improvement in treatment prognosis, SMART syndrome may be increasingly observed in patients who have undergone cranial radiation therapy. Thus, it is critical that physicians be able to accurately diagnose SMART by correctly recognizing symptoms and by ruling out other possible causes of neurological manifestations.
